# Life Narratives Are More Other-Centered, More Negative, and Less Coherent in Turkey Than in Germany: Comparing Provincial-Turkish, Metropolitan-Turkish, Turkish-German, and Native German Educated Young Adults

**DOI:** 10.3389/fpsyg.2018.02466

**Published:** 2018-12-07

**Authors:** Neşe Hatiboğlu Altunnar, Tilmann Habermas

**Affiliations:** Department of Psychology, Goethe University Frankfurt, Frankfurt, Germany

**Keywords:** life narrative, life story, autobiographical reasoning, cultural life script, individualistic identity

## Abstract

An individualized and coherent life story has been described as the form of identity that is required by highly mobile individualistic Western societies, whereas more family-oriented, traditional societies require more role-based, synchronic identities. Therefore in individualistic cultures entire life narratives can be expected to be more coherent and to contain more autobiographical arguments that contribute to life narrative coherence. This cultural group difference is expected to be mediated by individuals’ conformity to their respective cultural normative concept of biography, such that more conformity leads to less life narrative coherence and fewer autobiographical arguments. We tested these expectations by eliciting entire life narratives and cultural life scripts from four different cultural groups of students of technical universities: from provincial Karabük and from metropolitan Istanbul in Turkey, as well as from students with a Turkish migrant and with a native German background from urban Frankfurt am Main, Germany (*N* = 96). Expectations were confirmed for global life narrative coherence and autobiographical arguments with self-event connections. Conformity with a normative concept of biography indeed partially mediated cultural influences on life narrative coherence. Life narratives from Turkey also contained more family-related events and, unexpectedly, were more negative. Thus creating a coherent life narrative is more typical for cultures that require autonomous, individualized selves rather than for cultures requiring more related selves, reflecting the life story’s suitability for expressing individualized identities and its lesser suitability for expressing interdependent identities.

## Introduction

The life story is a form of identity which binds together heterogeneous life events to define the self and establish self-continuity and purpose in life (Erikson, 1968; McAdams, 2001). The integrative potential of a coherent life story makes it a form of identity specifically adapted to modern societies with highly mobile and individualized life courses and independent selves. Therefore it is of interest to explore whether the use of the integrative potential of the life story varies between cultures and subcultures depending on how independent versus interdependent individuals are expected to be. We first introduce the life story and its empirical qualities, then discuss conceptions of cultural differences on the dimension of independence-interdependence and their possible relation to the life story, to finally present two channels through which cultures may influence the life story.

Before we develop our argument by relating the life story to individualistic versus interrelated cultures, we need to introduce some qualities of life stories and the corresponding theoretical concepts and technical terms. Erikson (1968) described the life story as the modern form of identity which first develops in adolescence. McAdams (2001) accordingly defined identity as an internalized and evolving story of the self which is created by individuals and shaped by the culture they are embedded in. The individual life story ties together past, present, and future by providing unity and purpose. Thereby it helps to maintain a sense of identity across situations and over time (McAdams, 1988).

The life story is most completely manifested in entire life narratives. At any one point in time, an individual has one life story, but may produce somewhat differing life narratives (texts) depending on the communicative situation. Life narratives allow empirical access to individuals’ life stories. Life narratives differ from a mere collection of event-specific stories by requiring the stories to cohere so that they communicate individuals’ personal development and therefore reflect their identities. The more life narratives are globally coherent, the better they integrate specific events and aspects of the self with each other. We introduce four aspects of life story coherence as well as autobiographical arguments which are instrumental in creating coherence. Our aim is to specify how life narratives reflect personal continuity across change, which we believe renders them the ideal form of identity for highly mobile, individualistic societies.

Habermas and Bluck (2000) defined four aspects of global coherence of life narratives. The first aspect is created by adherence to *a cultural concept of biography*, a standard biography including a list of typical life events with normative timing, which is also termed *life script* (Berntsen and Rubin, 2004). *Temporal coherence* refers to how events are narrated in chronological order so that listeners are oriented when in life events took place. *Causal-motivational coherence* refers to how narrators create causal and motivational relations between distant events as well as between these and the self and its development. These links explain how narrators became who they are at present. *Thematic coherence* refers to overarching themes common to heterogeneous events, thereby integrating them within the life narrative (Habermas and Bluck, 2000).

An important means to create coherence in life narratives is *autobiographical arguments*. They form links among parts of one’s life as well as between these and the self and its development in an attempt to integrate one’s personal past and present (Habermas and Bluck, 2000). Autobiographical arguments can be found in entire life narratives as well as in more limited single event narratives. Some autobiographical arguments link events to the narrator’s personality, termed *self-event connections*. These may explain actions and events by reference to stable personality traits, thereby *maintaining stability*. Or they explain change in personality and insights gained in specific life experiences, thus *engendering change*, as does also a mixed group of o*ther autobiographical arguments* (Pasupathi et al., 2007; Habermas, 2011; Köber et al., 2015).

A coherent life narrative is expected to demonstrate some stability of the narrator’s personality, but also development and change. These two aspects of diachronic identity are reflected by stability-maintaining and change-engendering autobiographical arguments, respectively, which in turn contribute to thematic and causal-motivational coherence, respectively (Köber et al., 2015). Change engendering self-event connections and other autobiographical arguments bridge personal change by creating a developmental story, thus creating self-continuity across change.

These two aspects of life story integration correspond to the two strategies for maintaining personal identity over time, one based on self-sameness across time, the other on narrative self-continuity across change (Chandler et al., 2003; Habermas and Köber, 2015b). Sameness across time can be maintained under stable social circumstances, whereas highly mobile social contexts require engaging in change-engendering autobiographical reasoning which creates self-continuity across change (e.g., Thorne et al., 2004; Pals, 2006; Habermas and Köber, 2015a).

Having introduced the various aspects of life narratives, we now turn, secondly, to those socio-cultural forces that appear to require individuals to elaborate a diachronic identity, i.e., a life story, and their historical and cross-cultural variation. It is not clear whether the subjective life story is a universal phenomenon (Tonkin, 1992). However, the development of Western individualism has been linked to the rise of a biographical conception of identity as represented by literary autobiography and individual developmental life narratives, superseding a more synchronic identity defined by social relationships and positions (Giddens, 1991). The diachronic, biographical conception of identity is better adapted to continuously changing relationships and positions which do not follow a clearly predefined route. Thus in the modern Western world, the self is conceived as a reflexive, individualized project, or as a narrative that continuously needs to be updated and rewritten to achieve self-continuity across change (McAdams, 2001). In socio-cultural contexts that require flexibility and individualized life-courses, life narratives need to be causally motivationally coherent to bridge change, which is supported by change-engendering autobiographical arguments. In contrast, socio-cultural contexts that define individuals by stable social relations and positions, these relationships may be more important for defining identity than the individual life story (Wang, 2016). Therefore in these socio-cultural contexts life stories may not need to be as causally motivationally coherent and require fewer change-engendering autobiographical arguments.

Cultures have been typified with a dichotomy of Western individualism and an independent self versus Eastern collectivism and interdependent self (Markus and Kitayama, 1991). The dichotomy has been criticized as too undifferentiated (e.g., Schwartz, 1990; Matsumoto, 1999; Vignoles et al., 2016). For example, Kağıtçıbaşı (2005) attempted to better capture within-culture variations and effects of migration with a tripartite model linking cultural family models to respective modal person concepts. The interdependence family model, typical for collectivistic cultures, rural regions, and low SES, has an authoritarian/dependency oriented child rearing style. The resulting related self is sensitive about group loyalty, conformity, and maintaining family rules. The independence family model, common among individualistic cultures and urban areas with a permissive parenting style favors developing an autonomous self, which is characterized by well-defined self-boundaries and following one’s own rules. Finally the emotional interdependence family model common in urban regions of collectivistic cultures and ethnic minority groups from collectivistic cultures in the Western world emphasizes autonomy and close emotional connectedness between generations in child-rearing practices. The resulting autonomous-related self is both autonomous and still to a certain degree sensitive to group loyalty and emotional expectations of parents (Kağıtçıbaşı, 2005).

Individualization appears to show not only in an independent person concept, but also in the individualization of the life course. In the European context, a preponderance of individualistic (versus interdependent) values as well as of a more flexible, individualized life course can be found in North-Western compared to South-Eastern European countries (Nico, 2014).

Finally, we highlight two channels through which society and culture influence life stories: normative cultural conceptions of the life course and culturally formed reminiscing practices, sustaining an interdependent versus independent orientation. First, the life course itself is shaped by society’s institutional and economic structure through determining the timing of schooling and retirement (Kohli, 2007; Mayer, 2009). In addition, life narratives build upon cultural master narratives (McLean and Syed, 2016) and cultural concepts of biography (Habermas and Bluck, 2000). The latter contains culturally conceived life phases (Thomsen, 2015; Arnett, 2016) and a set of transitional events with age norms attached to them, termed *life script* (Berntsen and Rubin, 2004). This script serves as a guide for planning and evaluating life, as a framework for recalling important autobiographical memories, and for selecting biographically salient events for inclusion in the life story. Life scripts from different countries such as Turkey (Erdoğan et al., 2008; Hatiboğlu and Habermas, 2016), Australia (Janssen and Haque, 2018), Denmark (Berntsen and Rubin, 2004), Germany (Habermas, 2007), the United States (Rubin et al., 2009; Coleman, 2014), Japan (Janssen et al., 2014), the Netherlands (Janssen and Rubin, 2011), Malaysia (Haque and Hasking, 2010; Janssen and Haque, 2018), Qatar (Ottsen and Berntsen, 2013) and China, Mexico, and Greenland (Scherman et al., 2017) provide strong evidence for the existence of cultural life scripts with reasonable cultural and religious differences.

Second, cultures shape individuals’ selves by engaging them in different narrative practices geared toward developing the critical skills needed to be a competent member of that culture, which in turn influence the development and structure of individual autobiographical memory (Fivush, 2011; Wang, 2016). The goals of the cultural self-concept affect autobiographical memory in terms of its accessibility, style, and content (Conway and Pleydell-Pearce, 2000). Compared to individualistic Western cultures, personal narratives of children and adults in Eastern interdependent cultures focus more on themes of community and morality than on themes of autonomy and achievement. They tell less detailed personal narratives and focus more on group norms and group needs and less on their own activities, thoughts, and feelings (Pillemer, 1998; Leichtman et al., 2003). Easterners also describe themselves more in terms of relationships than of personal attributes (Wang, 2006, 2016; Göz et al., 2017). As expected by Kağıtçıbaşı (2005), these differences do not only show between Western and Eastern countries, but also within countries, for instance between Western and Eastern Turkey (Sahin-Acar and Leichtman, 2015).

Summing up, individualistic, highly mobile Western cultures require creating coherent life narratives as primary form of identity which allows an individualized and flexible way of securing self-continuity, whereas in interdependent, traditional Eastern cultures coherent life narratives may be less important relative to more synchronic, contextualized forms of identity defined by relationships to others. Accordingly, we expected life narratives to be less coherent in interdependent subcultures, and to specifically have less causal-motivational coherence.

Two of three relevant studies support this expectation, Chen et al. (2012) and Reese et al. (2017) found that New Zealand adolescents of European descent embedded turning point memories more in their lives by pointing out earlier causes or motives and later consequences (causal-motivational life story coherence) than adolescents of Maori and Chinese descent. In contrast to our expectations, Dunlop and Walker (2015) found fewer change-related other autobiographical arguments and more statements about self-stability in entire life narratives by 28 Canadian students of European descent than in 24 students who had immigrated from Asia. However, the focus on change in Asian immigrants’ life narratives may not reflect cultural differences but might have resulted from their recent experience of migrating. Finally, a recent cross-sectional questionnaire study with a large sample covering many different countries provided first evidence that a culture’s belief that identity was defined by social context and roles (interdependent self) was related to defining personal identity across time by self-sameness, whereas other cultures (independent self) preferred to base personal identity in time on constructing self-continuity across change in life narratives (Becker et al., 2018).

The present study shows several advantages over the studies just mentioned. It compares people living in different cultures, compares immigrants’ offspring with non-immigrant offspring living in the same country as well as in their parents’ home country, and it compares within-country differences between urban and rural areas. This study investigated differences between Kağıtçıbaşı (2005) three categories (with two groups in the middle category: provincial interdependent culture, migrants from interdependent to individualistic culture and urban interdependent culture, independent culture) in terms of global coherence of and autobiographical reasoning in entire life narratives. Furthermore, the present study actually measured cultural differences at the individual level by using an individual measure of interrelatedness (versus individualism) close to the construct measured, conformity with the respective group’s cultural life script.

This study tested two hypotheses. Individualistic cultures as well as urban life style consider the self and the life story as a project which needs to be worked on. Therefore, the cultural expectation to fashion an individualized identity by creating more coherent life narratives and using more change-engendering autobiographical reasoning should be higher in individualistic and urban than in interdependent and rural cultures. Hypothesis 1 thus predicts less global life narrative coherence, less change-engendering and more stability-maintaining autobiographical reasoning in provincial Turkey (interdependence) than in urban Turkey, than in Turkish migrants’ offspring in Germany (emotional interdependence), than in urban Germans (independence).

Earlier we had found in a larger sample of the same four groups as used in this study, and including the present sample, that the individually named cultural life scripts conformed more with the respective group’s shared cultural life script the more interdependent (vs. individualistic) the group is according to Kağıtçıbaşı’s model (2005): Life script conformity was thus highest in provincial Turkey, next in metropolitan Turkey and in Turkish-Germans, and lowest in Germans (Hatiboğlu and Habermas, 2016). To test in this study whether the group differences in life narratives predicted by Hypothesis 1 are mediated by individual conformity, we used the similarity of the individual life script with the respective group’s shared cultural life script as an indicator of cultural conformity. Hypothesis 2 thus predicts that individual conformity with the cultural life script mediates the influence of group, so that the higher conformity is, the lower life narrative coherence and change-engendering autobiographical reasoning will be. This would explain at least part of the group differences predicted in Hypothesis 1. Because this is the first cross-cultural study of entire life narratives, we also explore group differences in the content of life narratives.

## Materials and Methods

### Participants

Participants were four groups of students from universities for applied sciences studying a variety of subjects such as social work, elderly care, informatics, child development, information technology, textile technology. The provincial Turkish group was from Karabük, a medium-sized provincial center situated at the Black Sea. Participants had been living with their parents in nearby rural areas before starting to study at the faculty for applied sciences in Karabük. The urban Turkish group comprised young adults born in Istanbul, whose parents or grandparents had migrated to Istanbul from various rural regions of Turkey. The third group comprised Germans of Turkish descent, i.e., second generation Turkish-Germans living in Frankfurt am Main and born in Germany. The fourth and last group consisted of German participants with German parents also living in Frankfurt. Participants were approached via flyers in universities of applied sciences both in Germany (University of Applied Sciences Frankfurt) and Turkey (Karabük University, Faculty of Applied Sciences; Istanbul University, Faculty of Applied Sciences). Participants received 20€ or the Turkish equivalent as compensation.

Each of the four groups was composed of 24 participants (12 males and 12 females) between the ages of 20 and 30 (total *N* = 96). Age did not differ significantly between groups, *F*(3,92) = 0.121, *p* = 0.94, $\eta_{p}^{2}$ = 0.004, with mean ages 23.12 years (*SD* = 2.13) for Karabük, 23.29 (*SD* = 2.36) for Istanbul, 23.50 (*SD* = 2.12) for Germans of Turkish descent (in brief: Turkish Germans), and 23.25 (*SD* = 2.13) for Germans of German descent (in brief: Germans). However, their parents’ mean years of education did differ significantly, *F*(3,92) = 19.35, *p* = 0.00, $\eta_{p}^{2}$ = 0.387, with the shortest education in Karabük (*M* = 7.31, *SD* = 3.01), next Istanbul (*M* = 9.00, *SD* = 3.02), then Turkish Germans (*M* = 10.95, *SD* = 3.24), and the longest parental education in the German group (*M* = 13.45, *SD* = 2.42), reflecting different mean levels of education in the respective reference groups.

### Procedure

All interviews were conducted by the first author, who is a native Turk from Istanbul and speaks German fluently. They were conducted in a room assigned by the respective Turkish university or by Goethe University Frankfurt. Turkish German participants were free to choose the language they felt most comfortable with, half of them choosing German, the other half Turkish. Following Berntsen and Rubin (2004), participants were asked to provide a life script by imagining an ordinary infant of their own gender and cultural background, to write down the seven most important events that were most probably to take place in their life, and to estimate a culturally expected age for each event. Following Habermas and de Silveira (2008), participants were then asked to write down the seven most important personal memories from their own life and to order them chronologically. Then we asked them to narrate their life story within 15 min and to integrate these seven memories into their life narrative.

Life narratives were recorded and transcribed verbatim. The text was divided into propositions, which were defined as all comprehensible main or subordinate clauses. Two bilingual coders independently divided sixteen life narratives balanced for sex and group into propositions, agreeing on 96.6% of them. The remaining life narratives were divided into propositions by one coder. Reading the life narratives, raters/coders could not help but identify the group membership of the narrator. One of the two raters/coders was blind to the hypotheses, whereas the other (the first author) was not.

### Material

#### Global Life Narrative Coherence

Life narratives were rated for global coherence with manuals already used in an earlier longitudinal study (cf. Köber et al., 2015) (Table 1). Each of the scales was defined in a paragraph and by brief anchor definitions for each of the respective seven points. *Temporal coherence* shows how well a life narrative provides an overall temporal orientation to listeners. *Causal-motivational coherence* is indicated by the developmental consequentiality of events; the scale measures the degree to which the reader understands how past experiences are integrated into a developmental pathway of the narrator’s personality and life. *Thematic coherence* refers to how much diverse individual elements of a life narrative are thematically connected. Raters were trained by the original rater and co-author of the scale, Isabel Peters. All life narratives were rated independently by the first author and one other rater. Average measure intraclass correlations based on all life narratives were *r_ic_* = 0.85 for causal-motivational coherence, *r_ic_* = 0.78 for thematic coherence and *r_ic_* = 0.72 for temporal coherence.

**Table 1:** Ratings of types of global life narrative coherence.

Type of global coherence	Definitions
Temporal coherence	1 - One can never tell when and in what order something occurred. 2 - One can almost never tell when and in what order something occurred. 3 - One can often not tell when and in what order something occurred. 4 - One can partly tell when and in what order something occurred, but partly one cannot. 5 - One can mostly tell when and in what order something occurred. 6 - One can almost always tell when and in what order something occurred. 7 - It is always crystal clear when and in what order something occurred.

Causal-motivational coherence	1 - No development of the personality at all becomes clear, merely superficial changes. 2 - No development of the personality becomes clear, merely changes in preferences, specific attitudes or habits. 3 - A development of the personality is claimed but not substantiated or a development of personality is described but not designated as such. 4 - A development of the personality is basically substantiated by the events described, but it is not elaborated upon. 5 - The development of the personality becomes clear on the whole through some of the events described, however, not to a full extent. 6 - The development of the personality becomes clear through some of the events described, but is not always comprehensible down to the smallest detail. 7 - The development of the personality becomes clear in its turning-points and its motives.

Thematic coherence	1 - Between the individual episodes narrated no connection is discernible. 2 - Only one theme is addressed, which is why the connection between the episodes is not abstract but concrete. 3 - With some episodes differing in content it is possible to recognize a common motive, theme or a thematic category. 4 - Through episodes heterogeneous in content, thematic or motivational similarities can be clearly discerned, which are not, however, explicitly named. 5 - There is a basic attempt to establish a connection between heterogeneous episodes, between most episodes, however, the connection remains unclear or is exclusively implicit. 6 - Between many heterogeneous episodes a connection is established, but in part the connection remains unclear or is exclusively implicit. 7 - Between the various heterogeneous episodes there is established a connection in a logical and comprehensible fashion.


#### Autobiographical Arguments

We coded self-event connections, which link personality or personal values to specific life events. We differentiated change engendering from stability maintaining self-event connections. *Change-engendering self-event connections* include explanations of change in personality/values by specific events and revelations of unknown personality aspects by specific events. *Stability maintaining self-event connections* include explanations of events/actions by personality and the discounting of an event as being atypical for personality (cf. Pasupathi et al., 2007; Habermas, 2011). We also coded six *other autobiographical arguments*: developmental status, biographical background, lesson learned, generalized insights, formative experience, turning points which also deal with change (cf. Habermas and Paha, 2001; Habermas and de Silveira, 2008; for the manuals see footnote 1^1^).

Both from a theoretical standpoint and empirically (Köber et al., 2015), the six other autobiographical arguments and the change-engendering self-event connections bridge change in life and contribute to causal-motivational coherence, whereas stability-maintaining self-event connections contribute to thematic coherence (see Table 2). Coders were trained by the second author, who had originally written the manual. All life narratives were coded independently by the first author and one other coder. Cohen’s Kappas based on all participants were *K* = 0.85 both for other autobiographical arguments and for self-event connections. Hypotheses were tested using the frequency of specified arguments relative to the total number of propositions.

**Table 2:** Types of autobiographical arguments.

Stability-maintaining self-event connections
**Personality/value/talent is explains or shows in a typical behavior/event**
The personality trait/value/talent is exemplified by an event/action, or the personality trait/value/talent motivates or explains the action/event *I was always such an extremely shy child, so I like almost never said anything, and there were thirty of us, and the problem was that somehow they all knew each other and I was the only girl who really didn’t know anyone*.
**Atypical action/event in spite of personality/value/talent**
The link between a trait/value/talent and event shows an action not typical of the person, or the person’s refraining from a typical action.
*When suddenly the child plops out and you have it in your arms and that was actually the last time when I really remember when I shed tears so I normally try to control myself but in that case everything like ca- came out*.

**Change-engendering self-event connections**

**Event explains change in personality/value/talent**
Through an event the person changes his/her personality, values or talents. The change is deep reaching and lasting and not simply situative.
*This trip, I think, changed an awful lot for me so for one thing at that moment I understood what people mean by “the meaning of life is living” and it just made me a bit more self-aware*.
**Event reveals/leads to the recognition of personality traits, values or talents**
Through an action/event the narrator becomes aware of a hitherto hidden trait/value/talent. The trait/value/talent already existed before the event. Thus the event does not change the self but only the perception of the self.
*It took me a long time just to admit for starters that I want somebody and that I would like a woman I like, I’d dealt with it for a long time, but underneath the desire as always there, and this new therapist was in a position to bring this desires to the light of day*.

**Other autobiographical arguments**

**Developmental status**
The narrator makes assumptions about a specific developmental status and corresponding abilities, interests, etc.
*At the time I wasn’t aware of any of that, after all I was still too young for it*.
**Biographical background**
A person’s behavior or experience is explained with the help of earlier experiences and circumstances in that person’s life that have created a special sensitivity toward certain situations or lent them a particular significance.
*And when he came so threateningly toward me I completely blew my top, knocking him to the ground*.
*Only later did it occur to me that perhaps the reason for it was that he reminded me of the man who raped me*.
**Formative experiences**
Explicit claim that circumstances and experiences of a person’s life have had a lasting, formative influence on this person.
*Girlfriends have had a strong impact on me*.
*Yes, perhaps it has led to me no longer attaching so much importance to money today*.
**Lessons learned**
The narrator draws a lesson from a narrated experience. The narrator has learnt something for the future, how s/he should handle certain, relatively specific situations in the future.
*After that I told myself, next time I fall in love, I must take care that school doesn’t suffer from that*.
**Generalized insight, life maxims**
Universally valid insights into and realizations about how “the world” or “life” generally function. Included in this code are also maxims.
*You don’t get on in life, if you don’t use your elbows*.
*I missed him for many months*. *It’s probably always like that, when it’s the first kiss*.
**Turning points**
Code statements about turning points in life, a change of direction in life, breaks or severe disruptions in life.
*The fact that all of a sudden the child was there turned my life upside down*.


#### Emotional Valence of Seven Most Important Personal Memories

All memories were coded as either negative, neutral, or positive by two coders. Average measure intraclass correlation was *r_ic_* = 0.97. Disagreements in all ratings and codings were resolved by discussion.

#### Life Script Conformity

To establish shared cultural life scripts for each group, we added 80 more participants to each group (half men; total *N* = 415; one German had missing data), who were sampled in the same way as the participants of this study. We averaged the 104 individual life scripts provided by each group to establish four shared cultural life scripts (for more details cf. Hatiboğlu and Habermas, 2016). We needed these cultural life scripts to calculate the conformity of each individual’s life script (the seven events named for a normal infant’s future life) with the respective group’s shared cultural life script. This score is termed *life script typicality score* (Bohn and Berntsen, 2008). It measures the agreement between what an individual believes to be a normal life and what the individual’s group believes to be a normal life in terms of probable life events. It was calculated for each individual by weighing each of the seven nominated life script events by its relative frequency (between 0 and 100) in the entire normative group (*N* = 104), summing them up and dividing them by the number of nominated life script events. Resulting scores can range from 0 to 100. High values indicate a high degree of conformity of the individual life script with the respective group’s shared cultural life script. Here, we use life script conformity as an indicator of interdependent (versus individualistic) orientation.

## Results

We start by describing differences in life narratives between groups. We first test group differences of coherence and autobiographical reasoning, to then test a possible mediating role of conformity, i.e., whether individual differences in conformity explain the group differences. With multiple dependent variables, we first ran a multivariate and then follow-up univariate tests with linear contrasts. We also report descriptive correlations. Finally we explore the impact of the valence of events in life narratives.

### Description of Life Narratives in the Four Groups

The number of propositions of life narratives did not differ significantly between groups, *F*(3,92) = 0.919, *p* = 0.435, $\eta_{p}^{2}$ = 0.029, with mean number of propositions 244.7 (*SD* = 46.90) for Karabük, 261.5 (*SD* = 70.13) for Istanbul, 254.5 (*SD* = 93.69) for Turkish Germans, and 277.5 (*SD* = 63.77) for Germans. The duration of life narratives did not differ either, *F*(3,92) = 1.041, *p* = 0.378, $\eta_{p}^{2}$ = 0.033. Mean duration was 14.11 min (*SD* = 1.76) for Karabük, 13.13 (*SD* = 1.66) for Istanbul, 13.81 (*SD* = 2.50) for Turkish Germans and 14.03 (*SD* = 2.40) for Germans.

To provide a sense of the qualitative differences between the four groups, we listed in Table 3 the frequency with which life events were named among the seven most important memories before being told as part of the life narratives. The kinds of most important life events support the idea that culture affects the way we tell our lives by influencing which events are deemed biographically salient to be included in the life story. For example, the Turkish groups from Karabük, Istanbul, and Frankfurt frequently mentioned life experiences involving interpersonal relations such as family quarrel, other’s death, health problems in the family, problems with friends, being supported by the family, family members’ problems with the law, importance of social relations, parents’ divorce, financial problems of the family, and spending childhood far from one’s family. For example, the percentage of family quarrel was 63% in Karabük, 54% in Istanbul, 25% in Turkish Germans, and only 17% in Germans. Moreover, the following events were never once mentioned by the German group: other’s death, family support, importance of social relations, spending childhood far from family, legal, psychological and financial problems in the family.

**Table 3:** Relative frequencies (%) of life events among seven most important memories (ordered according to their frequency in the Karabük group).

Event	Karabük	Istanbul	Turkish German	German
University	71	46	58	83
Family quarrel	63	54	25	17
Other’s death	50	08	30	–
Academic difficulties	38	46	21	17
Move	33	46	21	–
Begin school	33	37	38	41
Health problems in family	33	08	–	08
Family support^∗^	29	17	25	–
Problems with friends	25	21	13	–
Begin high school	21	33	25	33
Falling in love/First partner	21	29	38	63
Own birth	21	21	21	08
Serious disease/illness	21	08	21	08
Not so severe illness/accident	17	08	–	–
Personal psychological problems	17	–	–	08
Neglect/abuse experience	13	13	–	–
High school graduation	13	–	25	63
Legal problems in family	13	–	–	–
Importance of social relations	13	–	08	–
Parents’ divorce	08	08	17	13
Separation from family as child	08	08	17	–
Getting into fights	08	–	–	–
Hobbies/leisure activities	–	21	08	25
Psychological problems in family	–	21	–	–
First job	–	17	08	25
Financial problems in the family	–	17	–	–
Begin daycare	–	13	21	25
Having stepparents	–	13	–	–
Parent‘s death	–	13	–	–
First friend	–	13	–	–
First sex	–	13	–	–
Problems in romantic relations	–	08	08	–
Parents’ dating story	–	08	–	–
Secondary school			21	21
Traveling	–	–	13	38
Visiting/short term residence in Turkey	–	–	13	–
Marriage	–	–	08	–
Puberty	–	–	08	–
Circumcision	–	–	08	–
Finding own identity/religion	–	–	08	–
Leave home	–	–	–	42
First vacation without parents	–	–	–	17
Confirmation	–	–	–	13
Community service	–	–	–	13
Baptism	–	–	–	08
Other	67	79	79	71


*^∗^Family support refers to events during which the family supported the narrator emotionally or financially.*

On the other hand, an orientation toward individualism and autonomy was reflected in the exclusive nomination by Germans of leaving parents’ home and traveling without parents for the first time among the most important life events. Although the Karabük group did not list leaving home among the seven most important seven life events, the majority of participants mentioned in their life narratives that they had had to leave their parents’ home to go to university, describing it as a very painful experience. Most of them wanted to return to their parents’ home after graduating. This difference between the Karabük and the German group reflects that while in German culture leaving home is considered a positive developmental transition to adulthood, in rural Turkish culture it is seen as a stressful non-normative life event.

The religious rite de passage circumcision was specific for the Turkish German group, possibly reflecting the importance of religion in a bi-cultural context. However, also the German group mentioned religious rites de passage, baptism and confirmation. Additionally, although it was not reported among the most important life events, the conflicts between Turkish and German life style and the acculturation experiences were prominent in Turkish Germans’ life narratives. Interestingly, traveling and secondary school were the items shared only by the two Frankfurt groups among the most important memories. These commonalities may reflect the integration of two cultures for Turkish Germans and the role of the national educational system. Lastly, the number of education-related events indicates the importance of the educational system in all of these students’ lives.

### Hypothesis 1: Group Differences in Life Narrative Coherence and Autobiographical Arguments

**FIGURE 1:**
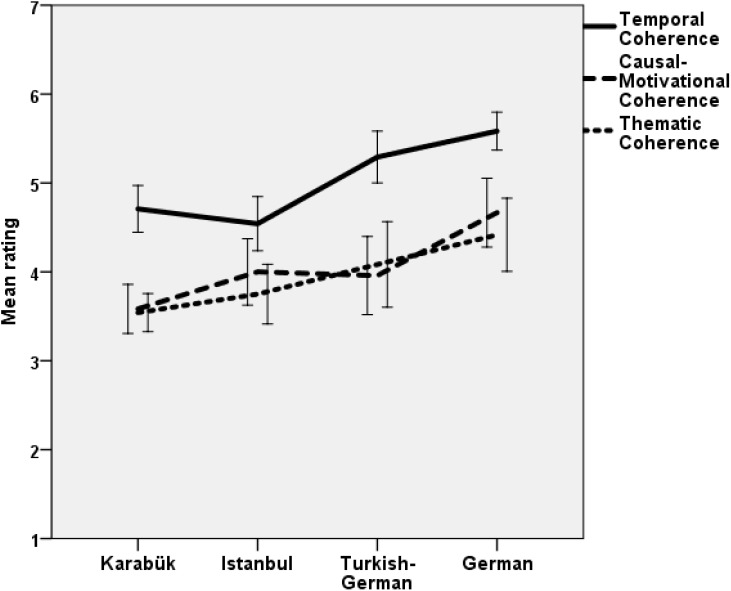
Mean life narrative coherence by groups with confidence intervals (95%).

We expected less global coherence and change related autobiographical reasoning in provincial Turks than in urban Turks and Turkish Germans, than in urban Germans, respectively. For global coherence, we first ran a MANOVA with all three aspects as dependent variables and group and gender as factors. The hypothesis was tested by a linear contrast with groups ordered in the sequence Karabük, Istanbul, Turkish Germans, Germans. The Istanbul group was positioned before the Turkish German group because the latter comprised not migrants, but migrants’ offspring. Possible gender differences were explored and will be reported only if significant. The multivariate linear contrast was highly significant, *F*(3,86) = 13.02, *p* < 0.001, η^2^ = 0.312, as was the undirected difference between groups, *F*(9,264) = 5.24, *p* < 0.001, η^2^ = 0.152. Univariate analyses confirmed the hypothesis for all three aspects of coherence, with significant linear contrasts for temporal coherence, *F*(1,88) = 34.45, *p* < 0.001, η^2^ = 0.281, for causal-motivational coherence, *F*(1,88) = 16.16, *p* < 0.001, η^2^ = 0.155, and for thematic coherence, *F*(1,88) = 13.44, *p* < 0.001, η^2^ = 0.133. Figure 1 shows that all effects were in the expected direction, Karabük students with the lowest and Germans with the highest global coherence of life narratives.

**FIGURE 2:**
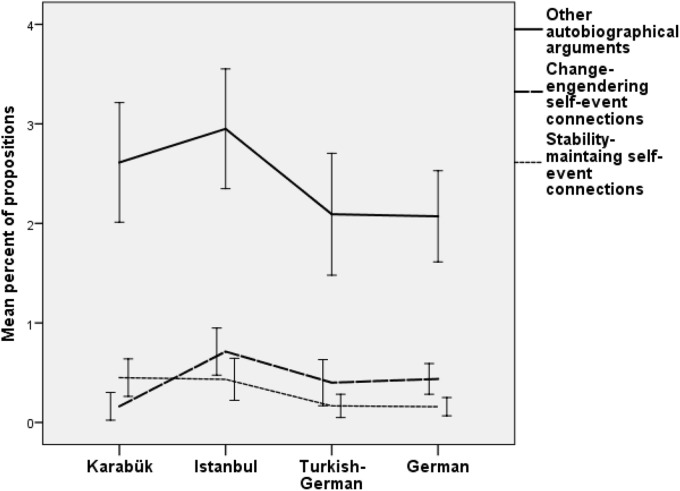
Mean percentage of propositions with other autobiographical arguments, change engendering and stability maintaining self-event connections by groups with confidence intervals (95%).

For the second part of Hypothesis 1 regarding autobiographical reasoning we expected change-engendering self-event connections and other autobiographical arguments to increase, but stability maintaining self-event connections to decrease between the groups from Karabük, Istanbul, Turkish Germans, and Germans. Again we first ran a MANOVA for all three kinds of autobiographical arguments and group and gender as factors. The multivariate linear contrast was significant, *F*(3,86) = 7.77, *p* < 0.001, η^2^ = 0.213, as was the undirected difference between groups, *F*(9,264) = 5.49, *p* < 0.001, η^2^ = 0.158. Gender also made a significant difference, *F*(3,86) = 6.04, *p* = 0.001, η^2^ = 0.174. Univariate linear contrasts confirmed the hypothesis only for stability maintaining self-event connections, *F*(1,88) = 12.89, *p* = 0.001, η^2^ = 0.128 (*r* = -0.35). The linear contrast for change-engendering self-event connections pointed in the expected direction (*r* = 0.16; Figure 2) without reaching significance, *F*(1,88) = 3.33, *p* = 0.07, η^2^ = 0.036. Finally, opposite to the expected direction, other autobiographical arguments decreased significantly between Karabük, Istanbul, Turkish Germans, and Germans, *F*(1,88) = 5.39, *p* = 0.023, η^2^ = 0.058 (*r* = -0.23). Univariate tests also showed that males (*M* = 2.68, 95% *CI* [2.30, 3.07]) used more other autobiographical arguments than women (*M* = 2.09, 95% *CI* [1.74, 2.440]), *F*(1,88) = 5.56, *p* = 0.021, η^2^ = 0.059, and also more change-engendering self-event connections (*M* = 0.53, 95% *CI* [0.37, 0.69]) than women (*M* = 0.27, 95% *CI* [0.17, 0.37]), *F*(1,88) = 10.03, *p* = 0.002, η^2^ = 0.106.

In our life story theory, we postulate that autobiographical arguments are instrumental in creating life narrative coherence, change-engendering arguments supporting primarily causal-motivational coherence and stability-maintaining arguments supporting thematic coherence (Habermas and Bluck, 2000; Habermas, 2011). This was confirmed in a German developmental sample (Köber et al., 2015) in which causal-motivational coherence correlated with all three kinds of autobiographical arguments, and thematic coherence with stability-maintaining self-event connections. In the present study, however, we could only replicate the correlation between causal-motivational coherence and change-engendering arguments. In addition thematic coherence correlated with change-engendering arguments, and temporal coherence negatively with stability maintaining self-event connections (see Table 4). Comparison of within-group correlations generally showed patterns consistent with the across-group correlations. Only the group of Germans resembled the earlier findings in a different German sample, causal-motivational coherence correlating both with other autobiographical arguments and change-engendering self-event connections. This speaks for cultural influences on these correlational patterns.

**Table 4:** Correlations of life narrative coherence with autobiographical arguments, change-engendering, and stability-maintaining self-event connections.

	Temporal coherence	Causal-motivational coherence	Thematic coherence
Autobiographical arguments	-0.14	0.11	-0.02
Change engendering self-events	0.13	0.52^∗∗^	0.30^∗∗^
Stability maintaining self-events	-0.30^∗∗^	-0.07	-0.01


*^∗∗^*p* < 0.01; ^∗^*p* < 0.05.*

### Hypothesis 2: The Mediating Role of Life Script Conformity

We used the life script typicality score as a measure of individual conformity with cultural norms regulating how to live a life, which characterizes an interdependent self in collectivistic cultures (Kağıtçıbaşı, 2005). By adding life script conformity as an additional continuous predictor to the model that tested Hypothesis 1, we tested how much individual conformity actually correlated with life narrative coherence as well as how much this reduced the predictive power of group membership. This would signal the expected role of conformity as mediating the influence of group differences.

As expected, conformity correlated negatively with all three aspects of life narrative coherence (Table 5). Correlations with autobiographical reasoning were as expected negative for change-engendering arguments, but near-absent for the other two arguments. Within-group correlations showed the same pattern. We tested a possible mediating role of conformity (Hypothesis 2) only for the dependent variables with a substantial correlation with conformity.

**Table 5:** Correlations of life script conformity and life narrative negativity with life story coherence, and autobiographical arguments, change-engendering, and stability-maintaining self-event connections.

	Temporal coherence	Causal-motivational coherence	Thematic coherence	Autobiographical arguments	Change engendering self-event connections	Stability maintaining self-event connections
Life script conformity	-0.37^∗∗^	-0.45^∗∗^	-0.35^∗∗^	-0.00	-0.35^∗∗^	0.14
Life narrative negativity	-0.40^∗∗^	-0.16	-0.05	0.15	-0.06	0.19


*^∗∗^*p* < 0.01; ^∗^*p* < 0.05.*

In a MANOVA with the three kinds of coherence as dependent variables, life script conformity as a continuous predictor, and group and gender as factors, conformity significantly predicted coherence, *F*(3,85) = 3.88, *p* = 0.012, η^2^ = 0.120, in addition to group, which still had a significant multivariate linear contrast, *F*(3,85) = 5.67, *p* < 0.001, η^2^ = 0.167, as well as a significant undirected effect, *F*(9,261) = 3.64, *p* = 0.001, η^2^ = 0.111. Univariate analyses confirmed the influence of conformity for two of the three aspects of coherence, *F*(1,87) = 5.20, *p* = 0.002, η^2^ = 0.056 for temporal coherence, *F*(1,87) = 10.29, *p* < 0.001, η^2^ = 0.106 for causal-motivational coherence, and a clear trend for thematic coherence, *F*(1,87) = 3.87, *p* = 0.051, η^2^ = 0.043. Linear contrasts for the effect of group on temporal coherence were *F*(1,87) = 15.70, *p* < 0.001, η^2^ = 0.153, on causal-motivational coherence, *F*(1,87) = 3.83, *p* = 0.053, η^2^ = 0.042, and on thematic coherence, *F*(1,87) = 4.71, *p* = 0.033, η^2^ = 0.051. Thus when adding conformity as a predictor, the multivariate effect size of the linear effect of group on coherence went down from η^2^ = 0.312 to about half, η^2^ = 0.167, and effect sizes of univariate linear group effects went down to almost half for temporal, and down to almost a quarter of the earlier sizes for causal-motivational and thematic coherence. Thus group differences in life narrative coherence were substantially, but not totally mediated by life script conformity.

A parallel ANOVA with change-engendering self-event connections as dependent variable showed a significant influence of conformity, *F*(1,87) = 7.83, *p* = 0.005, η^2^ = 0.083. The linear group contrast was not significant, *F*(1,87) = 0.03, *p* = 0.862, η^2^ = 0.000, while the undirected effect of group remained significant, *F*(3,87) = 7.88, *p* < 0.001, η^2^ = 0.214, showing that the earlier trend of a linear group effect was entirely mediated by conformity. Thus cultural conformity, as measured by life script typicality, clearly served as a mediator between group and life narrative coherence and change-engendering self-event connections, leaving some of the linear group effect on coherence to be accounted for.

### Exploring the Role of Negative Events in Life Narratives

To explore the unexpected results regarding group differences in other autobiographical arguments and their missing links with life narrative coherence, we conducted a series of exploratory analyses. They were based on the idea that a higher proportion of negative life events might require more autobiographical reasoning in the attempt to integrate them into the life story without necessarily succeeding yet. Such an initially still unsuccessful increase in autobiographical reasoning was termed attempt at meaning making by Park (2010). To check our coding of the valence of memories, we compared it with the valence rated by other participants in the larger sample (cf. Hatiboğlu and Habermas, 2016). Most events were evaluated similarly. Differences were noticeable regarding moving, which we coded as positive throughout, but was rated ambivalently by Germans and as mostly neutral to positive by Turkish participants, and regarding leaving home, which was rated positively by Germans, but ambivalently by the three Turkish groups. However, in this study moving was not named by the Germans, and leaving home was named almost exclusively by the Germans, so that these differences probably did not affect the group differences in negativity.

**FIGURE 3:**
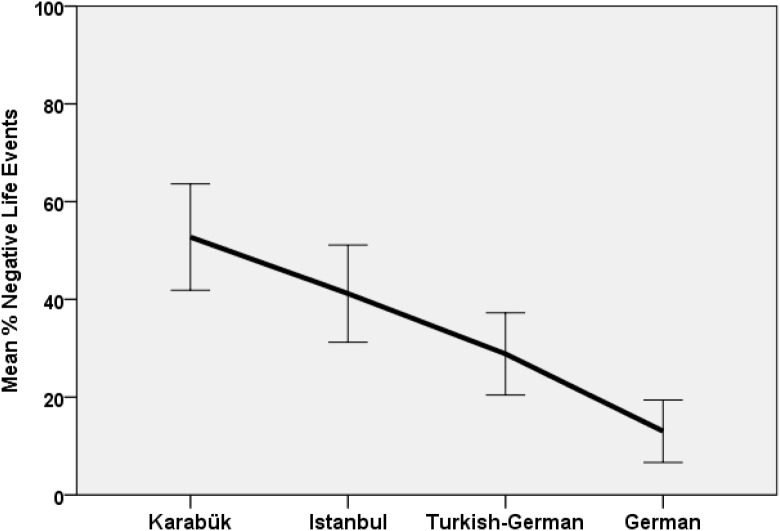
Life narrative negativity as mean percentage of negative life events among the seven most important memories by groups with confidence intervals (95%).

We then tested group and gender differences in the proportion of negative events among the seven most important own life events with an ANOVA, resulting in a strong group difference, *F*(3,88) = 13.87, *p* < *0*.001, η^2^ = 0.321. The Karabük group selected the greatest number of negative events and the Germans the smallest (see Figure 3).

To explore whether more negative events evoked higher degrees of autobiographical reasoning as a part of the process of working through negative events, we calculated correlations (Table 5). The proportion of negative events correlated only minimally with any of the autobiographical arguments. Only temporal coherence correlated significantly and negatively with the negativity of the seven most important memories. To test the relative weight of conformity and negativity for explaining group differences in temporal coherence, we ran an ANOVA with group and gender as the two factors and conformity and negativity as continuous predictors. Negativity did not contribute significantly, and group and conformity remained significant predictors.

## Discussion

The aim of this study was to take a closer look at the role of cultural and subcultural differences in life narrative coherence and autobiographical arguments and at their relation to the degree of cultural conformity. It is the first study of life narratives across cultures and subcultures, following calls for the cultural contextualization of psychosocial identity in the narrative identity tradition (Seaman et al., 2017). We will first discuss results regarding life narrative coherence and autobiographical arguments and the mediating role of life script conformity, then possible reasons for and consequences of the more frequent mentioning of negative events in Turkish life narratives, to finally note limitations and spell out implications of our findings.

### Life Narrative Coherence and Autobiographical Arguments

We had expected individualistic socio-cultural contexts to require more individualized life stories which therefore require more efforts to integrate change into a globally more coherent life narrative. The main hypothesis regarding cultural differences in global coherence of life narratives as well as the mediating role of individual cultural conformity were confirmed. Global coherence increased between groups from provincial Turkey, metropolitan Turkey, and Turkish migrants’ offspring, and Germans in urban Germany. This was partially mediated by cultural conformity as measured by how typical the individual life scripts were for the respective group’s cultural life script. Importantly, this findings support the widely held, but hitherto untested assumption that “in modern life, constructing one’s own meaningful life story is a veritable cultural imperative” (McAdams, 2001, p. 115). These findings support the notion that individualization of the life course and of identity require the creation of more globally coherent life narratives and favors the diachronic form of identity, the life story, over the synchronic form of social identity as defined by roles (cf. Becker et al., 2018).

Hypotheses regarding cultural differences in the degree of autobiographical reasoning were confirmed or supported by a strong trend for both kinds of self-event connections. The mixed group of other autobiographical arguments, however, showed a linear trend opposite to the expected direction. In fact, the highest proportion of any kind of autobiographical arguments was used by participants from Istanbul. Corresponding to the somewhat diverging results for life narrative coherence and autobiographical reasoning, only one of the expected correlations between the coherence and autobiographical arguments was confirmed, namely between causal-motivational coherence and change-engendering self-event connections.

A possible explanation for the unexpected high frequency of other autobiographical arguments in the Istanbul and Karabük groups could be the high frequency of negative life events. Self-relevant losses lead to identity disruption (Papa and Lancaster, 2016). Negative life events (McLean, 2008) and identity disruptions (Habermas and Köber, 2015b) call for change-related autobiographical reasoning in order to integrate them into the life story. Thus the higher amount of negative events in the Karabük and Istanbul groups might have led to more autobiographical reasoning without yet leading to a full integration of them into the life narrative, because processing and integrating negative experiences requires time and emotional distance from them (Park, 2010; Habermas and Berger, 2011; Habermas, 2019). This interpretation, however, was not supported by this study, because the proportion of negative events in life narratives did not correlate with other autobiographical arguments.

The peak of autobiographical reasoning in the Istanbul group may result from the impact of a historical event. The Gezi Protest was a common theme not among the most important memories, but in the life narratives. Data collection in Turkey took place some months after the Gezi Protest, a movement with strong student participation which started May 28, 2013. They were initially directed against urban development plans for Istanbul’s Taksim Gezi Park, and may have instigated autobiographical reasoning.

### Cultural Variations in the Negativity of Life Narratives

So why do life narratives from Turkey contain more negative events than those from Germany? Possibly everyday life is more comfortable in Germany because the welfare state better buffers existential risks like illness and disoccupation. This might suggest an alternative explanation for the cultural differences in global coherence, namely that it is easier to create global coherence if life is easier, because negative events are non-normative and therefore more difficult to integrate into a life narrative than normative events. However, the closely knit support system of Turkish families may compensate or even outweigh the German welfare system in providing support for the contingencies of life.

Anyway the high frequency of negative life events in Turkish groups need not reflect an actually higher frequency of negative events in Turkish than in German families. Taking into account the interdependent cultural background, family-related events play a central role in Turkish life style and were named more frequently. They tend to be more non-normative and therefore negative because only the narrators’ own normative life events help structure the life story and are therefore frequently mentioned, but not the normative life events of significant others. Accordingly, positive autobiographical memories are mostly about the self, whereas most important negative autobiographical memories are almost as often about others as about the self across cultures (Scherman et al., 2015). Thus the frequency of negative events may reflect the central role of family and close relationships rather than differences in the actual frequency of such events. However, it is more difficult to create a coherent life narrative, especially a coherent developmental story, if it encompasses more others’ and more negative life events, because they do not conform to the cultural concept of biography and need to be actively integrated into the life narrative. This explanation resonates with Markus and Kitayama’s (1991) reasoning that an interdependent self-construal requires more situational adaptation to others and therefore requires less self-consistency. Related findings have led to the coining of the term dialectical self-concept (Spencer-Rodgers et al., 2009).

Both explanations fit results from cross-cultural research on well-being, which is influenced positively both by gross national income as well as by individualistic self-construal (Cheng et al., 2016). The inclusion of more family-related life events reflects a stronger familial, interdependent orientation as well as the lower importance given to fashioning a coherent, highly individualized life story in that culture.

### Limitations and Implications

Cultural differences between groups covaried with differences in parents’ educational level. Given the differences in the general educational level between the four socio-cultural groups and the influence of formal education on attitudes and the life course, this covariation is probably intrinsic to the cultural differences studied. Nevertheless it would be of interest to compare the possible influence of level of education on the life story between cultures. The moderate number of interviewees in each group limits the possibility to generalize from these findings. It is due to scarce resources in the face of the labor-intensity of collecting and analyzing narrative data. However, an earlier publication did show that our samples were typical for their respective groups (Hatiboğlu and Habermas, 2016). Still, both expected and unexpected results call for a replication, ideally with different subcultures.

Nevertheless, future studies should keep the strength of the present study by comparing not only students from different nations, but by comparing different subcultures. Other subcultures than native versus immigrant Germans and urban vs. provincial Turks should also be tested. Future studies should also measure the timing and severity of negative life events independent from life narratives. For example, problems in school might be evaluated as not as serious as the loss of a parent, so that these events require different degrees of autobiographical reasoning. Similarly, recent negative events could be associated with higher amounts of autobiographical reasoning and might still be more disruptive for life narrative coherence compared to older negative life events. Therefore it could be argued that negative events are an important negative influence on life narrative coherence and autobiographical reasoning. Thus the nature of the relation between negative events and life narratives should be further examined.

## Conclusion

The aim of this first simultaneous study of cross-cultural and within-cultural differences in life narratives was to investigate the relation between socio-cultural environment and life narrative coherence and autobiographical reasoning. The study confirmed that life narrative coherence and autobiographical reasoning with self-event connections is affected by sociocultural context in a predictable way: More coherence, more change-engendering, and fewer stability-maintaining autobiographical reasoning characterize more individualized (sub-) cultures. The group of other autobiographical arguments, however, did not show the expected effects. Secondly, conformity with the cultural life script plays a mediating role for cultural influences on life narrative coherence. Finally, the more interdependent (sub-) cultures included more negative events in their life narratives. Matching the more interdependent outlook, most of these regarded not the narrators themselves, but the relationship with or individual fortunes of family members.

The present study’s findings on cultural differences of entire life narratives provides evidence for the assumed importance of a coherent life story in highly individualized Western societies compared to more interdependently oriented Eastern cultures (McAdams, 2001). They complement and match findings on cultural differences in autobiographical memories of specific events found mostly between United States Americans and Chinese (Wang, 2016). This study demonstrated that narrative studies, like other fields in psychology, need to empirically investigate the role of culture for their constructs and findings.

## Ethics Statement

This study was carried protectout in accordance with the recommendations of Ethikkommission des Fachbereichs 05, Goethe Universität Frankfurt with written informed consent from all subjects. All subjects gave written informed consent in accordance with the Declaration of Helsinki. The protocol was approved by the above-mentioned ethics committee with letter 2012-50.

## Author Contributions

The study was designed by NA with the guidance of TH, data were collected, transcribed, and coded by NA, and statistical analyses as well as the writing of the manuscript were done by NA with the assistance of TH.

## Conflict of Interest Statement

The authors declare that the research was conducted in the absence of any commercial or financial relationships that could be construed as a potential conflict of interest.

## Footnotes

1 http://www.psychologie.uni-frankfurt.de/57321401/30_Coding-manuals

## References

[B1] Arnett J. J. (2016). Life stage concepts across history and cultures: proposal for a new field on indigenous life stages. *Hum. Dev.* 59 290–316. 10.1159/000453627

[B2] Becker M. Vignoles V. L. Owe E. Easterbrook M. J. Brown R. Smith P. B. (2018). Being oneself through time: bases of self-continuity across 55 cultures. *Self Identity* 17 276–293. 10.1080/15298868.2017.1330222

[B3] Berntsen D. Rubin D. C. (2004). Cultural life scripts structure recall from autobiographical memory. *Mem. Cogn.* 32 427–442. 10.3758/BF03195836 15285126

[B4] Bohn A. Berntsen D. (2008). Life story development in childhood: the development of life story abilities and the acquisition of cultural life scripts from late middle childhood to adolescence. *Dev. Psychol.* 44 1135–1147. 10.1037/0012-1649.44.4.1135 18605840

[B5] Chandler M. J. Lalonde C. E. Sokol B. W. Hallett C. (2003). Personal persistence, identity development, and suicide. *Monogr. Soc. Res. Child* 68 131–138. 10.1111/1540-5834.00231 12951783

[B6] Chen Y. McAnally H. M. Wang Q. Reese E. (2012). The coherence of critical event narratives and adolescents’ psychological functioning. *Memory* 20 667–681. 10.1080/09658211.2012.693934 22716656

[B7] Cheng C. Cheung M. W. L. Montasem A. (2016). Explaining differences in subjective well-being across 33 nations using multilevel models: universal personality, cultural relativity, and national income. *J. Pers.* 84 46–58. 10.1111/jopy.12136 25234240

[B8] Coleman J. T. (2014). Examining the life script of African-Americans: a test of the cultural life script. *Appl. Cogn. Psychol.* 28 419–426. 10.1002/acp.3000

[B9] Conway M. A. Pleydell-Pearce C. W. (2000). The construction of autobiographical memories in the self-memory system. *Psychol. Rev.* 107 261–288. 10789197 10.1037/0033-295x.107.2.261

[B10] Dunlop W. L. Walker L. J. (2015). Cross-cultural variability in self-continuity warranting strategies. *J. Lang. Soc. Psychol.* 34 300–315. 10.1177/0261927X14555873

[B11] Erdoğan A. Baran B. Avlar B. Taş A. C. Tekcan A. I. (2008). On the persistence of positive events in life scripts. *Appl. Cogn. Psychol.* 22 95–111. 10.1002/acp.1363

[B12] Erikson E. H. (1968). *Identity: Youth and Crisis.* New York, NY: Norton.

[B13] Fivush R. (2011). The development of autobiographical memory. *Annu. Rev. Psychol.* 62 559–582. 10.1146/annurev.psych.121208.131702 20636128

[B14] Giddens A. (1991). *Modernity and Self-Identity.* Stanford, CA: Stanford University Press.

[B15] Göz I. Çeven Z. Tekcan A. (2017). Urban-rural differences in children’s earliest memories. *Memory* 25 214–219. 10.1080/09658211.2016.1150490 26924547

[B16] Habermas T. (2007). How to tell a life: the development of the cultural concept of biography. *J. Cogn. Dev.* 8 1–33. 10.1207/s15327647jcd0801DUL1

[B17] Habermas T. (2011). “Autobiographical reasoning: arguing and narrating from a biographical perspective,” in *The Development of Autobiographical Reasoning in Adolescence and Beyond: New Directions for Child and Adolescent Development*, ed. Habermas T. (San Francisco, CA: Jossey-Bass), 1–17. 10.1002/cd.285 21387528

[B18] Habermas T. (2019). *Emotion and Narrative: Perspectives in Autobiographical Storytelling.* Cambridge: Cambridge University Press.

[B19] Habermas T. Berger N. (2011). Retelling everyday emotional events: condensation, distancing, and closure. *Cogn. Emot.* 25 206–219. 10.1080/02699931003783568 21432668

[B20] Habermas T. Bluck S. (2000). Getting a life: the emergence of the life story in adolescence. *Psychol. Bull.* 126 748–769. 10989622 10.1037/0033-2909.126.5.748

[B21] Habermas T. de Silveira C. (2008). The development of global coherence in life narratives across adolescence. *Dev. Psychol.* 44 707–721. 10.1037/0012-1649.44.3.707 18473638

[B22] Habermas T. Köber C. (2015a). Autobiographical reasoning in life narratives buffers the effect of biographical disruptions on the sense of self-continuity. *Memory* 23 664–674. 10.1080/09658211.2014.920885 24912017

[B23] Habermas T. Köber C. (2015b). “Autobiographical reasoning is constitutive for narrative identity: the role of the life story for personal continuity,” in *The Oxford Handbook of Identity Development*, eds McLean K. C. Syed M. (Oxford: Oxford University Press), 149–165.

[B24] Habermas T. Paha C. (2001). The development of coherence in adolescents’ life narratives. *Narrat. Inq.* 11 35–54. 10.1075/ni.11.1.02hab

[B25] Haque S. Hasking P. A. (2010). Life scripts for emotionally charged autobiographical memories: a cultural explanation of the reminiscence bump. *Memory* 18 712–729. 10.1080/09658211.2010.506442 20803371

[B26] Hatiboğlu N. Habermas T. (2016). The normativity of life scripts and its relation with life story events across cultures and sub-cultures. *Memory* 24 1369–1381. 10.1080/09658211.2015.1111389 26564986

[B27] Janssen S. M. Haque S. (2018). The transmission and stability of cultural life scripts: a cross-cultural study. *Memory* 26 131–143. 10.1080/09658211.2017.1335327 28585471

[B28] Janssen S. M. J. Rubin D. C. (2011). Age effects in cultural life scripts. *Appl. Cogn. Psychol.* 25 291–298. 10.1002/acp.1690 24701028 PMC3972131

[B29] Janssen S. M. J. Uemiya A. Naka M. (2014). Age and gender effects in the cultural life script of Japanese adults. *J. Cogn. Psychol.* 26 307–321. 10.1080/20445911.2014.892493

[B30] Kağıtçıbaşı C. (2005). Autonomy and relatedness in cultural context: implications for self and family. *J. Cross Cult. Psychol.* 36 403–422. 10.1177/0022022105275959 30152264

[B31] Köber C. Schmiedek F. Habermas T. (2015). Characterizing lifespan development of three aspects of coherence in life narratives: a cohort-sequential study. *Dev. Psychol.* 51 260–275. 10.1037/a0038668 25621758

[B32] Kohli M. (2007). The institutionalization of the life course. *Res. Hum. Dev.* 4 253–271. 10.1080/15427600701663122

[B33] Leichtman M. Wang Q. Pillemer D. P. (2003). “Cultural variation in interdependence and autobiographical memory,” in *Autobiographical Memory and the Construction of a Narrative Self*, eds Fivush R. Haden C. (Mahwah, NJ: Erlbaum), 73–97.

[B34] Markus H. R. Kitayama S. (1991). Culture and the self: implications for cognition, emotion, and motivation. *Psychol. Rev.* 98 224–253. 10.1037/0033-295X.98.2.224

[B35] Matsumoto D. (1999). Culture and self: an empirical assessment of markus and Kitayama’s theory of independent and interdependent self-construals. *Asian J. Soc. Psychol.* 2 289–310. 10.1111/1467-839X.00042

[B36] Mayer K. U. (2009). New directions in life course research. *Annu. Rev. Sociol.* 35 413–433. 10.1146/annurev.soc.34.040507.134619

[B37] McAdams D. P. (1988). *Power, Intimacy and the Life Story.* New York, NY: Guilford Press.

[B38] McAdams D. P. (2001). The psychology of life stories. *Rev. Gen. Psychol.* 5 100–122. 10.1037/1089-2680.5.2.100

[B39] McLean K. C. (2008). Stories of the young and the old: personal continuity and narrative identity. *Dev. Psychol.* 44 254–264. 10.1037/0012-1649.44.1.254 18194024

[B40] McLean K. C. Syed M. (2016). Personal, master, and alternative narratives: an integrative framework for understanding identity development in context. *Hum. Dev.* 58 318–349. 10.1159/000445817

[B41] Nico M. (2014). Variability in the transitions to adulthood in Europe: a critical approach to de-standardization of the life course. *J. Youth Stud.* 17 162–182. 10.1080/13676261.2013.805877

[B42] Ottsen C. L. Berntsen D. (2013). The cultural life script of Qatar and across cultures: effects of gender and religion. *Memory* 22 390–407. 10.1080/09658211.2013.795598 23663084

[B43] Pals J. L. (2006). Authoring a second chance in life: emotion and transformational processing within narrative identity. *Res. Hum. Dev.* 3 101–120.

[B44] Papa A. Lancaster N. (2016). Identity continuity and loss after death, divorce, and job loss. *Self Identity* 15 47–61. 10.1080/15298868.2015.1079551

[B45] Park C. L. (2010). Making sense of the meaning literature: an integrative review of meaning making and its effects on adjustment to stressful life events. *Psychol. Bull.* 136 257–301. 10.1037/a0018301 20192563

[B46] Pasupathi M. Mansour E. Brubaker J. R. (2007). Developing a life story: constructing relations between self and experience in autobiographical narratives. *Hum. Dev.* 50 85–110. 10.1159/000100939

[B47] Pillemer D. (1998). *Momenteous Events, Vivid Memories.* Cambridge, MA: Harvard University Press.

[B48] Reese E. Myftari E. McAnally H. M. Chen Y. Neha T. Wang Q. (2017). Telling the tale and living well: adolescent narrative identity, personality traits, and well-being across cultures. *Child Dev.* 88 612–628. 10.1111/cdev.12618 27637177

[B49] Rubin D. C. Berntsen D. Hutson M. (2009). The normative and the personal life: individual differences in life scripts and life story events among USA and Danish undergraduates. *Memory* 17 54–68. 10.1080/09658210802541442 19105087 PMC3042895

[B50] Sahin-Acar B. Leichtman M. D. (2015). Mother–child memory conversations and self-construal in Eastern Turkey. Western Turkey and the USA. *Memory* 23 69–82. 10.4135/9781526449603 25029222

[B51] Scherman A. Z. Salgado S. Shao Z. Berntsen D. (2015). Life span distribution and content of positive and negative autobiographical memories across cultures. *Psychol. Conscious.* 2 475–489. 10.1037/cns0000070

[B52] Scherman A. Z. Salgado S. Shao Z. Berntsen D. (2017). Life script events and autobiographical memories of important life story events in Mexico, Greenland, China, and Denmark. *J. Appl. Res. Mem. Cogn.* 6 60–73. 10.1016/j.jarmac.2016.11.007

[B53] Schwartz S. H. (1990). Individualism-collectivism: critique and proposed refinements. *J. Cross Cult. Psychol.* 21 139–157. 10.1177/0022022190212001

[B54] Seaman J. O. Sharp E. H. Coppens A. D. (2017). A dialectical approach to theoretical integration in developmental-contextual identity research. *Dev. Psychol.* 53 2023–2035. 10.1037/dev0000383 29094967

[B55] Spencer-Rodgers J. Boucher H. C. Mori S. C. Wang L. Peng K. (2009). The dialectical self-concept: contradiction, change, and holism in East Asian cultures. *Pers. Soc. Psychol. Bull.* 35 29–44. 10.1177/0146167208325772 19106076 PMC2811254

[B56] Thomsen D. K. (2015). Autobiographical periods: a review and central components of a theory. *Rev. Gen. Psychol.* 19 294–310. 10.1037/gpr0000043

[B57] Thorne A. McLean K. C. Lawrence A. (2004). When remembering is not enough: reflecting on self-defining memories in late adolescence. *J. Pers.* 72 513–541. 10.1111/j.0022-3506.2004.00271.x 15102037

[B58] Tonkin E. (1992). *Narrating Our Past: The Social Construction of Oral History.* Cambridge: Cambridge University Press.

[B59] Vignoles V. L. Owe E. Becker M. Smith P. B. Easterbrook M. J. Brown R. (2016). Beyond the ‘east–west’dichotomy: global variation in cultural models of selfhood. *J. Exp. Psychol.* 145 966–1000. 10.1037/xge0000175 27359126

[B60] Wang Q. (2006). Relations of maternal style and child self-concept to autobiographical memories in Chinese, Chinese immigrant, and European American 3-year-olds. *Child Dev.* 77 1794–1809. 10.1111/j.1467-8624.2006.00974.x 17107461

[B61] Wang Q. (2016). Remembering the self in cultural contexts: a cultural dynamic theory of autobiographical memory. *Mem. Stud.* 9 295–304.

